# Who is killing South African men? A retrospective descriptive study of forensic and police investigations into male homicide

**DOI:** 10.1136/bmjgh-2023-014912

**Published:** 2024-04-10

**Authors:** Richard Matzopoulos, Lea Marineau, Shibe Mhlongo, Asiphe Ketelo, Megan Prinsloo, Bianca Dekel, Lorna J Martin, Rachel Jewkes, Carl Lombard, Naeemah Abrahams

**Affiliations:** 1 Burden of Disease Research Unit, South African Medical Research Council, Cape Town, South Africa; 2 Division of Public Health Medicine, School of Public Health, Faculty of Health Sciences, University of Cape Town, Cape Town, South Africa; 3 Johns Hopkins University School of Nursing, Baltimore, Maryland, USA; 4 Gender and Health Research Unit, South African Medical Research Council, Cape Town, South Africa; 5 Institute for Lifecourse Development, Faculty of Education, Health & Human Sciences, University of Greenwich, London, UK; 6 Division of Forensic Medicine & Toxicology, Faculty of Health Sciences, University of Cape Town, Cape Town, South Africa; 7 Office of the Executive Scientist, South African Medical Research Council, Cape Town, South Africa; 8 Biostatistics Unit, South African Medical Research Council, Cape Town, South Africa; 9 Division of Epidemiology and Biostatistics, Department of Global Health, Stellenbosch University, Cape Town, South Africa; 10 Division of Social and Behavioural Sciences, School of Public Health, Faculty of Health Sciences, University of Cape Town, Cape Town, South Africa

**Keywords:** Global Health, Public Health, Descriptive study, Injury, Traumatology

## Abstract

Not much is known about the perpetrators of male homicide in South Africa, which has rates seven times the global average. For the country’s first ever male homicide study we describe the epidemiology of perpetrators, their relationship with victims and victim profiles of men killed by male versus female perpetrators. We conducted a retrospective descriptive study of routine data collected through forensic and police investigations, calculating victim and perpetrator homicide rates by age, sex, race, external cause, employment status and setting, stratified by victim-perpetrator relationships. For perpetrators, we reported suspected drug and alcohol use, prior convictions, gang-involvement and homicide by multiple perpetrators. Perpetrators were acquaintances in 63% of 5594 cases in which a main perpetrator was identified. Sharp objects followed by guns were the main external causes of death. The highest rates were recorded in urban informal areas among unemployed men across all victim-perpetrator relationship types. Recreational settings including bars featured prominently. Homicides clustered around festive periods and weekends, both of which are associated with heavy episodic drinking. Perpetrator alcohol use was reported in 41% of homicides by family members and 50% by acquaintances. Other drug use was less common (9% overall). Of 379 men killed by female perpetrators, 60% were killed by intimate partners. Perpetrator alcohol use was reported in approximately half of female-on-male murders. Female firearm use was exclusively against intimate partners. No men were killed by male intimate partners. Violence prevention, which in South Africa has mainly focused on women and children, needs to be integrated into an inclusive approach. Profiling victims and perpetrators of male homicide is an important and necessary first step to challenge prevailing masculine social constructs that men are neither vulnerable to, nor the victims of, trauma and to identify groups at risk of victimisation that could benefit from specific interventions and policies.

**WHAT IS ALREADY KNOWN ON THIS TOPIC**

- Most homicide victims in South Africa are men, but little is known about perpetrators.

**WHAT THIS STUDY ADDS**

- This study describes the epidemiology of perpetrators and their relationship with victims.

**HOW THIS STUDY MIGHT AFFECT RESEARCH, PRACTICE OR POLICY**

- Recognising that men are vulnerable to trauma is a prerequisite for prevention. Identifying men at risk of victimisation will inform specific interventions.

## Introduction

South Africa (SA) has one of the world’s highest homicide rates with a substantial peak among young adult men,^1^ which is typical of countries and regions where homicide rates far exceed global averages.^2 3^ We know from qualitative research with men who rape women and with offenders incarcerated for violence that they are similarly influenced by a complex interplay of masculinities and dynamics within familial and intimate relationships, exposure to violence, untreated psychopathology and systemic institutional failures.^4–6^ Research indicates that a lower proportion of men are killed by family members than women, and that men account for a considerably greater share of homicides from ‘organised crime’ or denoted as ‘gang-related’. Gang violence may also be a localised contributor to violence between men in settings outside the home, particularly public spaces. However, the context of the crime for more than two-thirds of male homicide victims internationally is unknown, or ascribed to ‘other causes’.^3^ This limits our understanding of victim-perpetrator relationships and information needed for prevention.

In SA, very high levels of violence are influenced by social norms around the ready use of violence—a ‘culture of violence’—that is, strongly gendered, in that it is both predominantly perpetrated by men against men and reflected in the prevalent gender-based violence perpetrated by men against women. Gender norms also strongly influence aggression between men through the expectation that men compete for hierarchical positions with other men through physical fights over girlfriends, defending their honour by responding physically to slights or demonstrating strength and toughness as protectors of their families. Gender norms are likely to increase men’s resistance in the face of conflict as shown in a recent SA study of robbery-homicide. Although homicide during a robbery event remains rare, a male presence significantly increases the risk of co-occurring violence.^7^ In social settings, heavy episodic drinking that is prevalent among young adult men^8^ interacts with these norms to increase the possibility of more severe violence between men. The culture of violence also explains the ready use of violence by women reported in many contexts in SA, including in homes against children, in schools by teachers, in the community and in health facilities.^9 10^ However the perpetration of homicide by women has received little attention.^11^

The epidemiological profile of perpetrators of male homicide more generally is also under-researched. Nationally-representative studies on perpetrators were previously only undertaken for female and child homicides in 1999 and 2009^12–14^ until the SA Medical Research Council funded a comprehensive homicide study for 2017 that, for the first time, includes a male homicide module. We describe the epidemiology of male homicide with a specific focus on the perpetrator profile and their relationship with victims. We also compare the homicide victim profile of men killed by male versus female perpetrators.

## Methods

### Study design and sampling

This was a retrospective national study using a multistage stratified cluster sample. Our phase 1 sampling frame comprised 58 641 postmortem reports from 121 medicolegal laboratories (the primary sampling unit/cluster) operational in SA in 2017. For eight of SA’s nine provinces these laboratories were stratified by province and facility size: small (≤500 bodies), medium (501–1500 bodies) and large (>1500 cases). A second level of sampling was applied according to laboratory size. For small and medium laboratories all records were included. In large laboratories, except for child (<18 years) and adult female homicides, every second folder was selected. In total, 65 laboratories were selected with an expected sample of 22 733 records. These were complemented by all records from 16 laboratories in the Western Cape, where the health department maintains a surveillance system with compatible data. We applied analysis weights to account for the selection probabilities of laboratories within survey strata. Further detail on phase 1 sampling, fieldwork and data collection methods are provided elsewhere.^15^

A second sampling process included the identification of all men aged 18 years and older registered as a homicide, or injury death of undetermined intent. We randomly selected 20% of these deaths as cases for further investigation. We examined autopsy reports for each and used mandatory Crime Administration System (CAS) numbers to link cases with police investigations. If CAS numbers were incorrectly recorded, we used death register numbers and date of birth and death to link cases. We excluded cases with unknown sex or age (if uncertain whether >18 years) and where the cause of death was undetermined, for example, if the body was highly decomposed or recorded as skeletal remains.

### Data collection and variables

In phase 1, we collected data on age, (biological) sex, race (black, coloured, Indian/Asian, white or foreign national as recorded in postmortem and police reports), date of death, cause of death coded to the 10th International Statistical Classification of Diseases and Related Health Problems,^16^ CAS number, police station and autopsy samples (eg, blood alcohol levels) and forensic evidence (eg, bullets). The authors recognise that apartheid population group (or race) classifications are social categories that served a socio-historical political purpose. However, these classifications continue to be used in vital statistics and official health data and are considered an efficient and politically important way to monitor the persistence or reduction of social inequalities. Death register and death notification numbers were collected as identifiers for follow-up and to resolve data capture errors. Data were captured using KoboToolbox, a web-based data entry tool.

In phase 2, we interviewed police investigating officers, who examined case files to extract information on homicide events and investigations, including victim-perpetrator relationships where available. Interviews commenced with the verification of phase 1 data to link with crime investigation data using multiple variables (CAS numbers, identifiers, sex, age, cause and date of death). Victim data were complemented with additional information such as education and employment status. Interviews were conducted face-to-face whenever possible even during COVID-19 lockdown periods and telephonically when COVID-19 restrictions did not permit in-person contact, when police stations were too remote to justify travel costs or when specifically requested by the police. Perpetrators were defined as persons that the investigating officer considered responsible or suspected of committing murders. Perpetrators were considered ‘identified’ if there were coherent accounts of events pointing to culpability and ‘unidentified’ if there were no suspects or substantial doubts about culpability. In cases with multiple perpetrators, police were asked to identify the main perpetrator where possible. Identified perpetrators were then classified as ‘known’ or ‘unknown’. Known perpetrators comprised ‘family’ including direct and in-law relations and intimate partners, and ‘acquaintances’ including friends, colleagues and any other person known to the victim. Unknown perpetrators were strangers, including police or security personnel and victims of crime that acted in self-defence. Additional perpetrator information included age, sex, education, employment, known use of alcohol and drugs at the time of the event and firearm ownership.

### Statistical analysis

Descriptive statistics examined the distribution of all variables across the outcomes. We considered survey design, including the multistage structure of the data set, with weighting, stratification by laboratory size and as clusters. Where possible we estimated homicide rates within subgroups (family, acquaintance and stranger). For homicide rates by age and sex we used SA 2017 population estimates from a mathematical model.^17^ For population by race, employment status and setting we applied the 2013 race distribution from Dorrington,^18^ the proportion of employed adults aged 15–64 years from Statistics SA’s 2017 Quarterly Labour Force Survey^19^ and the housing geotype distribution (urban formal, urban informal and rural—farm or traditional area) from Statistics SA’s 2007 General Household Survey,^20^ respectively. We calculated age-standardised homicide rates using the world standard population^21^ with normalised weights to take into account the subgroup analysis of ages 18+ and age-specific homicide rates, and 95% confidence limits using standard methods for estimating CIs from complex multistage sample surveys (Taylor linearisation).

Data were analysed using Stata V.14.1.^22^ All analyses were conducted at the 5% significance level.

### Ethics

The Ethics Committee of the SA Medical Research Council approved the study (EC 008-5-2018). Further approval and data access were granted by National and Provincial Health Departments, Forensic Pathology and Police Services. We obtained written informed consent from investigating officers (electronically for telephonic interviews) before data were extracted. It was not appropriate or possible to involve patients or the public in our research.

## Results

**Figure 1:**
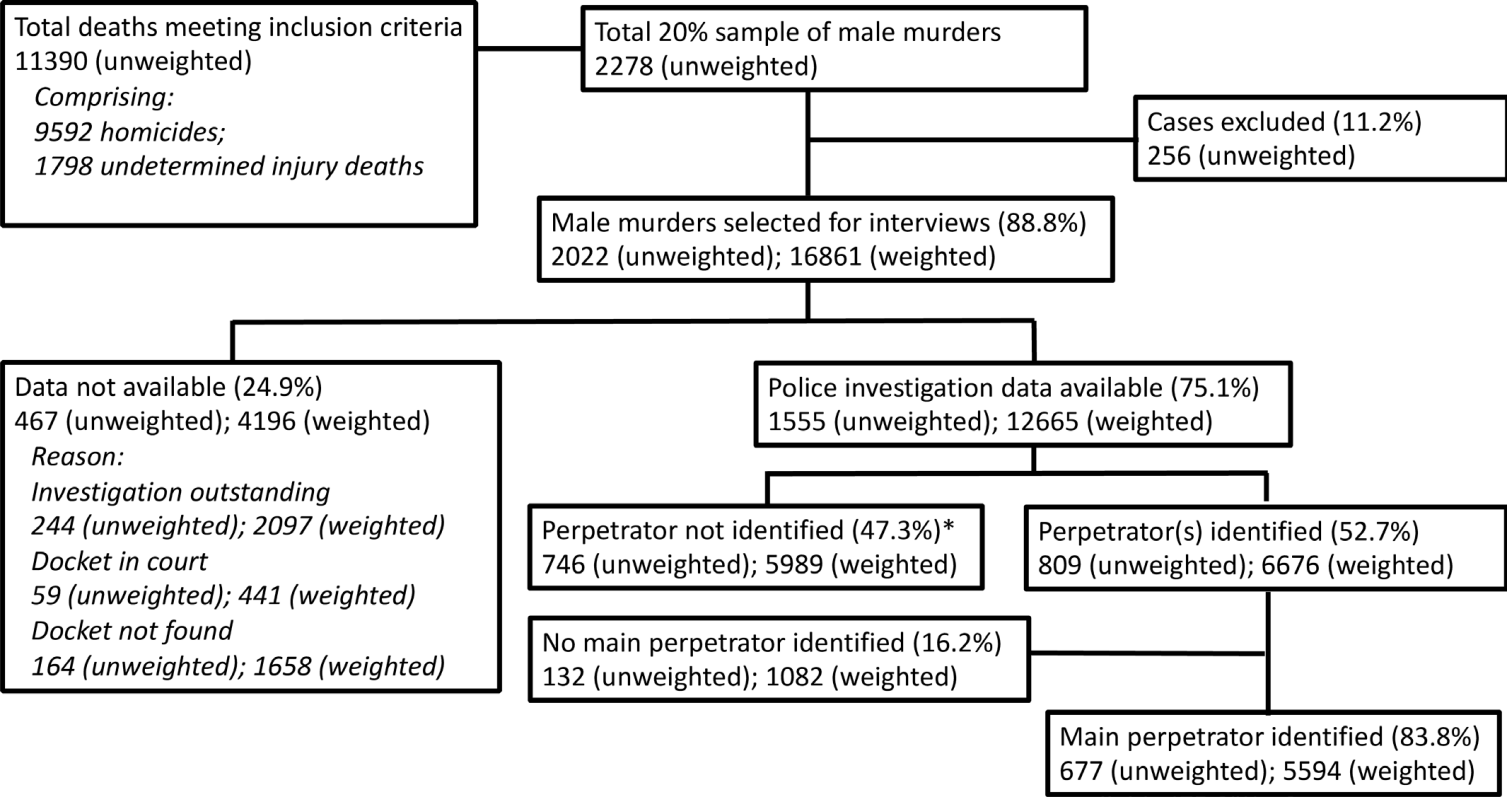
Flow diagram of the sample from 81 medicolegal mortuaries. * Includes 0,4% missing cases – i.e. 9 cases (unweighted); 54 (weighted)

Our sample of 22 822 deaths from eight provinces exceeded the expected sample by 89. After combining with 8174 Western Cape deaths we identified 11 390 cases meeting the inclusion criteria: men aged >18 years or adults of undetermined age, of which 9592 were classified homicide and 1798 injury deaths of undetermined intent. We selected 2278 homicide cases for follow-up interviews with investigating officers (20% of the total sample) after which we excluded a further 256 that they did not meet sampling criteria (eg, child, adult women). The remaining 2022 deaths sample represented an estimated 16 861 adult male homicides after weighting (figure 1).

We conducted interviews and accessed police investigation dockets for 1555 (75.1%) of these cases, which represented an estimated 12 665 murders after weighting. 805 (52%) interviews were conducted in person and 748 (48%) were telephonic. Of the 1555 cases for which investigation data were available, a perpetrator was known or suspected in 809 (52.7%), representing 6676 cases (after weighting). In 16.2% of cases, police were unable to identify a main perpetrator. The results that follow report weighted estimates for the subsample of 677 male homicides (5594 weighted) where information about perpetrators was known and a main perpetrator identified.

Stratifying cases by the relationship (family, acquaintance, stranger, unknown relationship) between the victim and perpetrator (online supplemental table 1) we found 63% of men were killed by an acquaintance at a rate of 17.8 per 100 000 population, 10% by a family member, 19% by a stranger. In 8% of cases the relationship was unknown. Men killed by family members were most often aged 30–44 years (40.9% of cases). Men aged 18–29 years were more likely to be killed at the hands of acquaintances and strangers, and where the victim-perpetrator relationship was unknown (52.6%, 45.8% and 60.3%, respectively). Homicide rates in these two age categories (18–29 and 30–44) were higher than in older ages across all victim-perpetrator relationship types, except when victims and perpetrators were family members.

10.1136/bmjgh-2023-014912.supp1 Supplementary data

Most men were killed with a sharp object whether by family, an acquaintance, a stranger, or person where the relationship was unknown (80.8%, 71.6%, 45.1% and 68.6%, respectively). Gunshot injuries were the second leading external cause of death across all victim-perpetrator relationship types, and were particularly common when perpetrators were strangers (43.8%).

Homicide rates among coloured men killed by family and acquaintances (7.0 (95% CI: 5.5 to 8.5) and 32.3 (95% CI: 28.1 to 36.4) per 100 000 population, respectively) were significantly higher than among African men (3.1 (95% CI: 2.4 to 3.8) and 21.1 (95% CI: 18.8 to 23.5)), which were in turn significantly higher than among whites (0.6 (95% CI: 0.0 to 1.5) and 0.0). African men were more likely than men of other racial groups to be killed by strangers (6.3 (95% CI: 5.6 to 7.1)), but this was only significant when compared with white men (2.8 (95% CI: 1.4 to 4.2)). Homicide rates among unemployed men were significantly higher than for employed men across all victim-perpetrator relationship types.

The highest rates of male homicide across all victim-perpetrator relationship types were recorded in urban informal areas. Rates of homicide by family members in urban informal areas (12.5 (95% CI: 8.6 to 16.3)) were significantly higher than in rural areas (4.2 (95% CI: 2.9 to 5.6)), which in turn were significantly higher than in urban formal areas (1.5 (95% CI: 1.1 to 2.0)). Men living in urban informal areas and rural areas (25.7 (95% CI: 19.5 to 31.9) and 23.6 (95% CI: 19.9 to 27.2), respectively) were significantly more likely to be killed by acquaintances than in urban formal areas (16.6 (95% CI: 14.8 to 18.5)).

Among men killed by family members, 79.6% occurred in the victim’s home. For all other relationship categories, public spaces such as roads, parks and shops/malls were the most common homicide locations, accounting for 49.1% of male homicides by an acquaintance, 60.3% by a stranger and 59.5% by a person whose relationship with the deceased was unknown. Recreational settings including bars and shebeens also featured prominently among men killed by acquaintances (23.1% of deaths).

Homicides were clustered around months coinciding with the Christmas and Easter festive periods in 2017 (January, April and December). More homicides were recorded on weekend days, including Fridays, but on Saturdays and Sundays in particular, across all victim-perpetrator relationship types.

Online supplemental table 2 presents characteristics of known perpetrators for the male homicide victims (n=5594) and is stratified by the relationship between the victim and the perpetrator. Over 90% of perpetrators who were acquaintances, strangers or where the relationship was unknown, were men. When the perpetrator was family, 55.8% were men and 44.2% were women. Perpetrators aged 15–9 years had the highest perpetrator rates regardless of relationship to the victim. Perpetrators identified as coloured accounted for significantly higher rates of homicide by family and acquaintances (2.9 (95% CI: 2.2 to 3.5) and 13.5 (95% CI: 12.6 to 14.6), respectively). Among foreign national perpetrators stranger homicides accounted for a larger share of deaths—11.0% compared with 8.1% of acquaintance and 3.1% of family killings.

10.1136/bmjgh-2023-014912.supp2 Supplementary data

Perpetrators were significantly more likely to be identified as unemployed than employed for male homicides involving family members (1.2 (95% CI: 0.9 to 1.6) vs 0.7 (95% CI: 0.5 to 0.9)) and acquaintances (8.6 (95% CI: 7.6 to 9.7) vs 4.1 (95% CI: 3.4 to 4.8)). Perpetrator alcohol use was reported in 40.7% of male homicides by family members and half (50.1%) of homicides by acquaintances. Drug use was less commonly reported, but was highest among cases where the perpetrators were identified as acquaintances (11.0%) or strangers (7.4%).

Perpetrators who were strangers had the highest likelihood of having a prior conviction (18.1%), followed by acquaintances (11.8%), when the relationship was unknown (10.3%) and family (5.1%). Multiple perpetrators were most common for homicide by strangers (21.7%) and where the perpetrator relationship was unknown (17.3%).

Of the 379 men killed by female perpetrators (online supplemental table 3), 96.0% of victims were known to the attacker (family members or acquaintances) and were family members in two-thirds of cases (66.2%). Most of these family members (90.0%) were intimate partners—226 cases in total. Almost half (49%) of the men killed by female family members, and 55% killed by female intimate partners, were aged 30–44 years. Acquaintances, which accounted for 29.8% of male homicides by a female perpetrator, were mostly aged 18–29 years (52.3%). Intimate partner homicides of men were most commonly perpetrated by women in the 30–44 years age category (43.4%), as were the killing of strangers (66.7%). Most male homicides at the hands of female perpetrators (85.5%) were the result of sharp force injuries and the use of firearms by female perpetrators was exclusively against intimate partners. Female perpetrators of intimate partner homicides and other family murders killed men in domestic settings (either the victim or perpetrator’s home), whereas public spaces accounted for a higher percentage of deaths when the female perpetrator was an acquaintance or stranger.

10.1136/bmjgh-2023-014912.supp3 Supplementary data

African men comprised the largest percentage of homicides across all relationship types for female perpetrators. Employed male intimate partners accounted for a larger share of homicides by female perpetrators (34.3%) than any other relationship type. Women were more likely to kill intimate partners and other family members in urban informal (47.6%) and rural areas (42.1%), whereas the killing of acquaintances by women was mostly concentrated in urban informal areas (53.3%). All white men killed by family members were killed by female intimate partners. Alcohol involvement was a feature of approximately half of female-on-male family and acquaintance murders. Female foreign national women were involved in a high percentage of homicides of male acquaintances (22.2%). No female perpetrators with prior convictions were suspected of perpetrating male homicides.

There was considerable variation in the victim-perpetrator relationships for male homicides depending on whether the perpetrator was female or male. Men accounted for 93% of the killing of non-intimate family members (parents, siblings, etc)—a significantly higher proportion than for female perpetrators (p<0.001). No male intimate partner homicides of male victims were recorded. Acquaintances (65.6%) also accounted for a significantly higher share of male homicides (p<0.001). For male perpetrators only family murders were concentrated in victims’ homes (77.6%), whereas public spaces were the most likely scene for deaths at the hands of a male acquaintance (48.9%) or unknown assailant (60.5%). Male perpetrators were also most likely to use sharp force to murder male victims (66.4% of cases) but were more likely to use firearms across all victim-perpetrator categories (p<0.003 for the family to p<0.001 for acquaintances and strangers). Gunshot injuries featured most prominently (36.8%) when the male perpetrator was not known to the victim. Male perpetrators were significantly more likely to kill unemployed male strangers (p<0.001) and acquaintances (p<0.016) than female perpetrators.

## Discussion

Our study indicates that in 2017 a man was killed by his female intimate partner in SA every 38 hours accounting for 4.1% of all male homicides. This is in stark contrast to female homicides in SA, where intimate partners account for more than half of all female homicides in studies spanning almost three decades from 1999 to 2017.^12 23^ This is somewhat lower than the available global estimate of 6.3% (95% CI: 3.1% to 6.35%) of male homicides committed by an intimate partner, but is within the range of the 95% CIs for the estimate. It is also within the range of estimates for the proportion of male homicides perpetrated by intimate partners in the African region (4.36% (95% CI: 1.56% to 6.47%),^24^ but these proportions do need to be considered in the context of SA having considerably higher homicide rates than global averages. Whereas our study recorded the intimate partner homicide of an estimated 226 men, a parallel study found that 1089 women were murdered by intimate partners in SA in the same year.^23^ This shows that not only was the risk to men from intimate partners less than a quarter the risk to women, but also that commonly reported intimate partner homicide rates are almost 25% higher when male intimate partner violence victims are included.

The vast majority of male homicides (63.1%) were at the hands of an acquaintance outside of the family but known to the deceased. Although there is a paucity of sex-disaggregated data describing victim-perpetrator relationships, this is considerably higher than has been recorded previously in the USA (35%),^25^ and in Johannesburg, SA among male adolescents (37%).^26^ This suggests that ‘stranger homicide’ that might typically co-occur during robbery events^3^—and which still occurs at a high rate compared with international measures at 5.4 per 100 000 population—is not a particularly strong driver of SA’s very high homicide rates. This is consistent with a recent analysis of police data in the context of robbery-homicide.^7^

Male homicide by an acquaintance was significantly higher in rural and informal urban areas than in formal urban areas. These deaths were overwhelmingly the result of sharp force injuries (71.6%) and the high percentage of cases in recreational settings, such as bars and taverns (23.1%), and the temporal pattern—60.9% on weekends and monthly peaks coinciding with major holiday periods—suggest that alcohol was an important contributing factor. These findings also align with research from several high-income countries that show an association between socioeconomic status and violence.^27–29^ In our study, the highest rates of male homicide occurred in informal urban areas, and unemployment was significantly associated with higher rates of male homicide across all victim-perpetrator categories.

Addressing male violence is central to preventing violence affecting men, women and children. Predominant social constructions of masculinity may lead to increased male violence.^30^ This undermines social safety and perpetuates an intergenerational cycle of violence in affected communities. Furthermore, common reactions to adverse events tend to differ between men and women, whereby men may cope more often using social withdrawal, anger, irritability, intimate partner violence and increased engagement in risk-taking behaviours.^31 32^ SA already has among the world’s highest levels of income inequality^33^ and informal settlements far from employment opportunities and social infrastructure are particularly affected. Even within low socioeconomic status communities violence rates are higher in areas with higher levels of deprivation.

These areas are also characterised by densely clustered informal alcohol retailers that expose residents to cheap and poorly regulated alcohol that further increases the risk of criminal and interpersonal violence.^34^ Patterns of harmful alcohol use are also a consequence of existing inequalities in socioeconomic systems—a legacy of SA’s apartheid dispossession.^35^ Harmful use was perpetuated by social and political systems of control such as the ‘dop’ system, which saw farm workers given alcohol as a benefit of employment,^36^ and access to alcohol was subject to strict regulation to ensure compliant migrant labour in the mining industry.^37^ The result is widespread harm, particularly in SA’s poorest communities with heavy episodic drinking a key risk factor for acute harms such as injuries.^38^ The effect of changing the pattern of drinking was recently demonstrated in SA when intermittent alcohol sales bans implemented as part of the COVID-19 response coincided with significant reductions in injury deaths and hospitalisations.^39–42^

While more than half of all male homicides occurred on weekends across all perpetrator types, this was most apparent when the victim was known to the perpetrator, that is, a family member or acquaintance. International data associate stranger homicide with criminal (eg, robbery) and collective (eg, gang and political) rather than interpersonal violence.^3^ This may explain the more frequent use of firearms among stranger homicides in our study (44%) than among cases where the victim was known to the perpetrator. Firearm use has been found to explain metro/non-metro differences in homicide risk^43^ and SA’s fluctuating homicide rates are influenced by its adherence to firearm control policies. Some 10 years after firearm homicides decreased significantly relative to non-firearm homicide coinciding with the implementation of SA’s Firearms Control Act of 2000,^7 44 45^ the increasing use of firearms has emerged as an important driver of increasing homicide rates. This has been ascribed to increased availability both through legal mechanisms and corruption,^44^ which recently led to class action litigation against the state by families affected by gunshot deaths after corrupt police officials redirected weapons marked for destruction to criminal gangs.^46^

With regard to perpetrator characteristics, the vast majority of male homicides overall (93%) were male-on-male encounters with the highest perpetration rates among men aged 18–29 years. This was most notable in relation to the murder of friends and acquaintances where perpetration by men aged 18–29 years was significantly higher than other ages. Male homicide by a family member (including intimate partners) affected older men, with the highest rates recorded in the 30–44 age category and accounted for the smallest share of male homicide.

Rates of family murder and acquaintance murder by perpetrators classified as coloured (3.0 per 100 000 population (95% CI: 2.3 to 3.6) and 13.4 per 100 000 population (95% CI: 12.4 to 14.4), respectively) were significantly higher than perpetrators classified as black (1.5 (95% CI: 1.2 to 1.8) and 8.6 (95% CI: 7.8 to 9.4)) and by perpetrators that were unemployed. This is consistent with research from 2009, which showed that homicide risk was higher for coloureds than blacks specifically in metro areas.^4^ Family and acquaintance murders were also characterised by a high percentage of alcohol use by perpetrators. A higher percentage of stranger homicides (21.7%) and homicides by unknown perpetrators (17.3%) involved multiple perpetrators. SA has the highest incarceration rates in Africa,^47^ but our study suggests high rates of recidivism among violent offenders. Perpetrators with criminal convictions accounted for 11.8% of all homicides and more than two-thirds (66.9%) involved the killing of friends and family. Perpetrators with criminal convictions accounted for a larger share (18.1%) of total stranger homicides than any other category.

A comparison of male and female homicide perpetration revealed that women were far more likely to kill known acquaintances, particularly intimate partners and other family members. Victims were also known to perpetrators for most male-on-male homicides, but the relationship was more distal, with acquaintances being the modal category and a substantial share of strangers as victims (28% compared with 4% for female perpetrators). This suggests that for female perpetrators the motivation for violence was overwhelmingly interpersonal, and within the domestic setting, rather than criminal and in the public space. This was emphasised by female perpetrators use of firearms—a weapon of choice for criminal violence—being used exclusively in the domestic setting. Whereas the larger share of male intimate partners and other family members killed by women in urban informal and rural areas may be expected due to possible links with deprivation and economic hardship, the high percentage of employed male intimate partner victims seems incongruous. This might be explained by some of these homicides being in self-defence in response to male entitlement or violence, but further research is needed.

For male perpetrators, almost two-thirds of victims were acquaintances, which is consistent with gender norms driving aggression between men and with alcohol acting as an accelerant. As well as increasing vulnerability to violence, as evidenced by SA studies of homicide cases in which more than half were alcohol positive,^48 49^ alcohol has also been shown to precipitate aggressive behaviour and is considered to be causally linked to interpersonal violence.^50–53^ Alcohol also features prominently among intimate partner homicide perpetrated by women (53% of cases). This may indicate that women drinking being more concentrated in the home. Men drinking is more ubiquitous across settings, but particularly common in low-income neighbourhoods where there is a proliferation of taverns and unregulated informal outlets. For example, more than 1000 outlets are located in Khayelitsha a large, low-income, high-violence neighbourhood of approximately 400 000 people. This equates to more than six times the recommended alcohol outlet density in California, one of few jurisdictions internationally where density limits are imposed, and up to 38 times that density limit in certain subareas within Khayelitsha.^34^

There are relatively few studies that explore the patterns of male homicide, and the impact of violence on male health specifically, despite men experiencing a disproportionate burden of homicide globally and in SA (United Nations Office on Drugs and Crime, 2019). Studies that stratify the homicide victim-perpetrator relationship among men vary. For instance, some studies stratify offenders by whether they are outside the family or within the family, dividing within the family between intimate partner/ex-partner and other family members.^54^ Other studies stratify the homicide victim-offender relationship at a more granular level, by whether the offender is an intimate partner, other family member, acquaintance or known person, stranger or the relationship is unknown.^25 55 56^ Many studies examining patterns of homicide victim-offender relationships do so by comparing victim gender and do not focus on men alone.^25 54–56^

This study has a number of limitations. The recorded date of death for homicides does not take into account the interval between the injury event and the date of death. Nevertheless, data from SA’s Western Cape suggest that for most homicides the death has followed within 8 hours of the injury event. That is because blood alcohol testing is performed routinely on all homicide cases except child deaths and deaths from injury sequelae more than 8 hours after the injury event. In the Western Cape 80% of homicide cases were tested for blood alcohol.^49^ With regard to alcohol as a risk factor for violence, the perpetrators’ alcohol level was not tested and instead we relied on victim alcohol levels to indicate alcohol in the context of homicides.

Furthermore, outside the Western Cape the proportion of cases with blood alcohol concentration was very low and some caution is therefore needed in interpreting the overall results. We have to assume some bias towards tests being requested in other provinces if there was suggestive evidence of alcohol involvement. The study presents a national profile of injury-related mortality by race, which in SA has provided a proxy for exposure to structural racism (and other racism-related exposures) due to the legacy of racial discrimination under apartheid and colonialism. Although almost 30 years after democratic elections, our study shows that inequalities still persist, at least in terms of homicide risk. We were unable to calculate homicide rates for foreign nationals due to the unavailability of reliable population information. Whereas some homicides by female perpetrators may have been due to self-defence, this would only be determined during court proceedings. The same may apply to homicides by male perpetrators. Moreover, in SA there has been an increase in contract killings^57^ with frequent recent media reports of female perpetrators of intimate partner homicide hiring hitmen.^58–61^ There may be more cases where this has happened that have not been discovered and so the number of female perpetrators may be an underestimate.

In conclusion, in-depth research on profiling victims and perpetrators has to date only been undertaken for female and child homicides in SA. While there is an urgent need to address violence against women and children, this needs to be integrated into an inclusive approach as set out in Sustainable Development Goals 16 and 5.2.^62^ In this study we examined the victim-perpetrator relationships of male homicide in SA with a specific focus on the victim and perpetrator characteristics as described in forensic and police records, with an additional analysis of a male homicide subgroup of particular interest: men killed by women. In documenting these considerable differences in rates of male homicide compared with female homicide we are not seeking to downplay or trivialise the very real problem of violence afflicting women in SA. A recent comparative risk assessment found that the overall burden was similar among men and women when the indirect effects of violence from exposure to intimate and non-partner violence, child maltreatment, sexual violence and abuse, community violence and bullying were taken into account.^63^ Instead we consider this to be an important and necessary first step to challenge the prevailing masculine social construct that men are neither vulnerable to, nor the victims of, trauma^64^ and to identify specific groups at risk of victimisation that could benefit from specific interventions and policies. For example, at a societal level we would recommend not only a redoubling of efforts to control alcohol and firearms, but also programmes to address the insidious effect of toxic masculinities and other societal norms that drive the excessive burden of physical violence borne by men in SA.

## Data Availability

Availability of data used in the study would be subject to permission by the Health Research Ethics Committee and provincial authorities that approved the original study. This is a recently completed study and the data set will initially be used for capacity development among the emerging researchers on study team. Thereafter access to a de-identified data set is available upon reasonable request. Requests should be sent to the convenor of the South African Medical Research Council’s Research Ethics Office, Ms Adri Labuschagne (Adri.Labuschagne@mrc.ac.za), for consideration. Guidelines for applications and related materials are available at: https://www.samrc.ac.za/research/rio-research-ethics-office A period of 24 months after publication of the main study results should elapse before requests are made, to allow the authors to publish substudies and further analyses.
