# Supplementary material for: Who is killing South African men? A retrospective descriptive study of forensic and police investigations into male homicide
Source: BMJ Glob Health. 2024 Apr 10;9(4):e014912. doi: 10.1136/bmjgh-2023-014912 (PMC11015244; doi:10.1136/bmjgh-2023-014912)
Supplement: Supplementary data [file bmjgh-2023-014912supp001.pdf]

Table 1. Victim characteristics of male homicides for selected covariates in South Africa in 2017 by victim-perpetrator relationship (n= 5594)

		Victim-perpetrator relationship*											
		Family			Acquaintance			Stranger			Unknown relationship		
Victim characteristics	Population	n (95% CI)	%	Rate per 100,000 (95% CI)	n (95% CI)	%	Rate per 100,000 (95% CI)	n (95% CI)	%	Rate per 100,000 (95% CI)	N (95% CI)	%	Rate per 100,000 (95% CI)
All adult males, 18+ years (n= 5594)	17,939,493	567 (458; 675)	10.1	3.0 (2.0; 4.0) **	3531 (3208; 3854)	63.1	17.8 (15.4; 20.1) **	1039 (927; 1152)	18.6	5.4 (4.2; 6.6) **	456 (330; 583)	8.2	2.3 (1.3; 3.3) **
Victim age group (n= 5574)		567 (458; 675)			3521 (3198; 3844)			1029 (913; 1146)			456 (330; 583)		
18-29	6,102,462	202 (162; 243)	35.7	3.3 (2.7; 4.0)	1854 (1681; 2026)	52.6	30.4 (27.5; 33.2)	472 (387; 556)	45.8	7.7 (6.3; 9.1)	275 (191; 360)	60.3	4.5 (3.1; 5.9)
30-44	6,401,940	232 (149; 315)	40.9	3.6 (2.3; 4.9)	1271 (1104; 1438)	36.1	19.9 (17.2; 22.5)	404 (336; 472)	39.3	6.3 (5.2; 7.4)	123 (77; 170)	27.0	1.9 (1.2; 2.7)
45-59	3,470,787	107 (66; 149)	18.9	3.1 (1.9; 4.3)	277 (222; 332)	7.9	8.0 (6.4; 9.6)	89 (44; 133)	8.6	2.6 (1.3; 3.8)	35 (2; 68)	7.7	1.0 (0.1; 2.0)
60+	1,964,304	25 (11; 39)	4.4	1.3 (0.6; 2.0)	120 (84; 156)	3.4	6.1 (4.3; 7.9)	65 (45; 85)	6.3	3.3 (2.3; 4.3)	23 (6; 40)	5.0	1.2 (0.3; 2.0)
External cause (n= 5594)		567 (458; 675)			3531 (3208; 3854)			1039 (927; 1152)			456 (330; 583)		
Sharp force	17,939,493	458 (339; 576)	80.8	2.6 (1.9; 3.2)	2530 (2237; 2823)	71.6	14.1 (12.5; 15.7)	469 (389; 549)	45.1	2.6 (2.2; 3.1)	313 (205; 421)	68.6	1.7 (1.1; 2.3)
Gunshot	17,939,493	56 (17; 94)	9.8	0.3 (0.1; 0.5)	511 (436; 585)	14.5	2.8 (2.4; 3.3)	455 (389; 520)	43.8	2.5 (2.2; 2.9)	98 (53; 143)	21.5	0.5 (0.3; 0.8)
Blunt	17,939,493	43 (18; 69)	7.7	0.2 (0.1; 0.4)	277 (216; 337)	7.8	1.5 (1.2; 1.9)	45 (17; 73)	4.3	0.3 (0.1; 0.4)	17 (0; 34)	3.7	0.1 (0.0; 0.2)
Other	-	10 (10; 10)	1.8	-	214 (141; 287)	6.1	-	70 (40; 101)	6.8	-	28 (11; 46)	6.2	-
Victim race (n= 5589)	-	567 (458; 675)		-	3526 (3203; 3849)		-	1039 (927; 1152)	-	-	456 (330; 583)	-	
African	13,931,072	434 (338; 531)	76.6	3.1 (2.4; 3.8)	2946 (2621; 3271)	83.5	21.1 (18.8; 23.5)	882 (780; 984)	84.9	6.3 (5.6; 7.1)	426 (300; 553)	93.4	3.1 (2.2; 4.0)
Indian/Asian	540,213	9 (0; 21)	1.6	1.7 (0.0; 3.9)	50 (36; 54)	1.4	9.3 (6.7; 10.0)	15 (1; 29)	1.4	2.8 (0.2; 5.4)	0	0.0	0.0 (0.0; 0.0)
Coloured	1,595,872	112 (88; 135)	19.7	7.0 (5.5; 8.5)	515 (449; 581)	14.6	32.3 (28.1; 36.4)	90 (58; 122)	8.7	5.6 (3.6; 7.6)	30 (30; 30)	6.6	1.9 (1.9; 1.9)
White	1,872,336	12 (0; 29)	2.1	0.6 (0.0; 1.5)	0	0	0.0 (0.0; 0.0)	52 (27; 78)	5.0	2.8 (1.4; 4.2)	0	0	0.0 (0.0; 0.0)
Undetermined	-				10 (0; 25)	0.3		0	0		0	0	
Other	-				5 (5; 5)	0.1		0	0		0	0	
Victim employment status (n= 5499)	-	567 (458; 675)		-	3479 (3158; 3800)		-	1007 (884; 1130)	-	-	446 (321; 572)	-	-
Employed	8,854,719	113 (72; 153)	19.9	1.3 (0.8; 1.7)	499 (400; 597)	14.3	5.6 (4.5; 6.7)	217 (162; 271)	21.5	2.5 (1.8; 3.1)	20 (0; 48)	4.5	0.2 (0.0; 0.5)
Unemployed	9,195,286	210 (140; 279)	37.0	2.3 (1.5; 3.0)	1617 (1391; 1843)	46.5	17.6 (15.1; 20.0)	376 (304; 448)	37.3	4.1 (3.3; 4.9)	87 (30; 144)	19.5	0.9 (0.3; 1.6)
Unknown		244 (177; 311)	43.1		1363 (1200; 1527)	39.2		415 (317; 513)	41.2		339 (252; 427)	76.0	
Setting (n= 5594)		567 (458; 675)			3531 (3208; 3854)			1039 (927; 1152)			456 (330; 583)		
Urban formal	10,374,409	159 (114; 204)	28.1	1.5 (1.1; 2.0)	1727 (1531; 1923)	48.9	16.6 (14.8; 18.5)	649 (554; 745)	62.5	6.3 (5.3; 7.2)	238 (165; 310)	52.1	2.3 (1.6; 3.0)
Urban informal	1,058,430	132 (91; 172)	23.2	12.5 (8.6; 16.3)	272 (206; 338)	7.7	25.7 (19.5; 31.9)	85 (51; 119)	8.2	8.0 (4.8; 11.2)	45 (12; 78)	9.9	4.3 (1.1; 7.4)
Rural	6,508,448	276 (189; 363)	48.7	4.2 (2.9; 5.6)	1533 (1293; 1772)	43.4	23.6 (19.9; 27.2)	305 (208; 401)	29.3	4.7 (3.2; 6.2)	174 (102; 246)	38.0	2.7 (1.6; 3.8)
Place of homicide (n= 5487)		567 (458; 675)			3491 (3152; 3830)			1032 (919; 1144)			397 (278; 517)		
Victim home	-	451 (347; 555)	79.6	-	416 (329; 503)	11.9	-	97 (58; 135)	9.4	-	88 (40; 136)	22.1	-
Perpetrator home	-	5 (5; 5)	0.9	-	92 (48; 136)	2.6	-	67 (28; 105)	6.5	-	0	0	-
Someone else home	-	23 (6; 40)	4.0	-	270 (191; 348)	7.7	-	75 (43; 107)	7.3	-	15 (15; 15)	3.8	-
Public space (road, park, shop,mall)	-	58 (25; 91)	10.2	-	1714 (1506; 1922)	49.1	-	622 (530; 713)	60.3	-	236 (148; 324)	59.5	-
Recreational setting (bar, shebeen***)	-	30 (10; 50)	5.3	-	805 (672; 938)	23.1	-	59 (36; 83)	5.8	-	33 (16; 51)	8.4	-
Other	-	0	0.0	-	195 (147; 242)	5.6	-	112 (82; 143)	10.9	-	25 (0; 53)	6.3	-
Month of Year of Homicide (n= 5594)		567 (458; 675)			3531 (3208; 3854)			1039 (927; 1152)			456 (330; 583)		
January	-	50 (22; 78)	8.8	-	212 (169; 255)	6.0	-	97 (63; 130)	9.3	-	42 (13; 71)	9.2	-
February	-	40 (15; 65)	7.1	-	196 (164; 228)	5.6	-	102 (64; 139)	9.8	-	18 (9; 26)	3.8	-
March	-	26 (3; 49)	4.6	-	391 (302; 480)	11.1	-	18 (9; 26)	1.7	-	12 (0; 29)	2.6	-
April	-	73 (32; 113)	12.8	-	352 (275; 430)	10.0	-	117 (80; 153)	11.2	-	99 (56; 141)	21.6	-
May	-	26 (12; 41)	4.7	-	169 (124; 214)	4.8	-	28 (11; 45)	2.7	-	39 (13; 65)	8.6	-
June	-	49 (22; 77)	8.7	-	220 (162; 277)	6.2	-	83 (59; 108)	8.0	-	58 (23; 93)	12.7	-
July	-	33 (4; 62)	5.9	-	320 (254; 385)	9.1	-	89 (65; 113)	8.6	-	15 (15; 15)	3.3	-
August	-	39 (20; 57)	6.8	-	266 (207; 325)	7.5	-	58 (25; 90)	5.5	-	53 (25; 81)	11.7	-
September	-	28 (19; 36)	4.9	-	300 (212; 388)	8.5	-	125 (80; 171)	12.1	-	28 (0; 57)	6.0	-
October	-	50 (4; 96)	8.8	-	288 (215; 362)	8.2	-	82 (46; 118)	7.9	-	20 (6; 35)	4.5	-
November	-	40 (15; 65)	7.1	-	298 (224; 372)	8.4	-	105 (73; 136)	10.1	-	12 (0; 29)	2.6	-
December	-	113 (85; 140)	19.9	-	520 (413; 627)	14.7	-	136 (82; 191)	13.1	-	62 (20; 104)	13.6	-
Day of Week of Homicide (n= 5594)		567 (458; 675)			3531 (3208; 3854)			1039 (927; 1152)			456 (330; 583)		
Monday	-	43 (20; 66)	7.6	-	302 (233; 371)	8.6	-	115 (75; 155)	11.1	-	17 (0; 34)	3.6	-
Tuesday	-	28 (3; 54)	5.0	-	193 (133; 253)	5.5	-	117 (78; 157)	11.3	-	25 (5; 46)	5.6	-
Wednesday	-	15 (15; 15)	2.7	-	261 (175; 347)	7.4	-	106 (40; 172)	10.2	-	20 (6; 35)	4.5	-
Thursday	-	34 (15; 54)	6.0	-	239 (186; 293)	6.8	-	48 (22; 73)	4.6	-	49 (12; 87)	10.8	-
Friday	-	100 (57; 143)	17.6	-	386 (318; 453)	10.9	-	73 (36; 109)	7.0	-	57 (18; 96)	12.4	-
Saturday	-	166 (113; 219)	29.3	-	1009 (828; 1191)	28.6	-	325 (245; 405)	31.3	-	156 (93; 220)	34.3	-
Sunday	-	180 (133; 227)	31.7	-	1141 (973; 1309)	32.3	-	256 (196; 316)	24.6	-	132 (87; 176)	28.8	-

* "Family" includes direct and in-law relations (biological father and mother, son, daughter, half-brother, half-sister, uncle, aunt, cousin, stepfather, stepmother, stepson, stepdaughter, foster father, foster mother, in-laws, child of boyfriend/girlfriend) and intimate partners (current or ex-spouses, cohabiting or dating partners, other sexual partners, or rejected suitors); "Acquaintance" includes a person known by sight (friend, neighbour, landlord, boarder/tenant, roommate, colleague/ co-worker, sexual rival [perpetrator romantically involved with victim's current or ex-wife/ husband/ girlfriend/ boyfriend], cell mate/inmate, employee, employer, gang member; and "Stranger" includes person unknown to victim and other includes security personnel, property owners (home, bottle store, shop), criminals, game ranger

** Age standardised rate per 100,000 population

*** Unlicensed tavern
