# Supplementary material for: Who is killing South African men? A retrospective descriptive study of forensic and police investigations into male homicide
Source: BMJ Glob Health. 2024 Apr 10;9(4):e014912. doi: 10.1136/bmjgh-2023-014912 (PMC11015244; doi:10.1136/bmjgh-2023-014912)
Supplement: Supplementary data [file bmjgh-2023-014912supp002.pdf]

Table 2. Perpetrator characteristics of male homicides for selected covariates in South Africa in 2017 by victim-perpetrator relationship (n= 5594)

		Victim-perpetrator relationship											
		Family			Acquaintance			Stranger			Unknown relationship		
Perpetrator characteristics	Population	n (95% CI)	%	Rate per 100,000 (95% CI)	n (95% CI)	%	Rate per 100,000 (95% CI)	n (95% CI)	%	Rate per 100,000 (95% CI)	N (95% CI)	%	Rate per 100,000 (95% CI)
All perpetrators, 15+ years (n= 5594)	40,163,034	567 (458; 675)	10.1	1.4 (0.8; 1.9) *	3531 (3208; 3854)	63.1	8.1 (7.0; 9.2) *	1039 (927; 1152)	18.6	2.4 (2.0; 2.9) *	456 (330; 583)	8.2	1.0 (0.7; 1.4) *
Perpetrator sex, 15+ years (n= 5594)		567 (458; 675)			3521 (3197; 3845)			1034 (922; 1147)			449 (323; 575)		
Male	19,295,712	316 (260; 372)	55.8	1.6 (0.8; 2.4) *	3408 (3111; 3706)	96.5	15.7 (13.6; 17.9) *	1024 (912; 1137)	98.6	4.9 (3.9; 5.9) *	444 (318; 570)	97.3	2.1 (1.4; 2.7) *
Female	20,867,322	251 (170; 331)	44.2	1.2 (0.6; 1.8) *	113 (50; 176)	3.2	0.5 (0.2; 0.8) *	10 (10; 10)	1.0	0.0 (0.0; 0.0) *	5 (5; 5)	1.1	0.0 (0.0; 0.0) *
Unknown	-	0	0.0	-	10 (0; 25)	0.3	-	5 (5; 5)	0.4	-	8 (0; 16)	1.6	-
Perpetrator age group, 15+ years (n=5130)		544 (439; 649)			3233 (2949; 3517)			929 (828; 1030)			424 (302; 545)		
15-29	14,856,508	253 (180; 327)	46.5	1.7 (1.2; 2.2)	1984 (1771; 2196)	61.3	13.4 (11.9; 14.8)	469 (405; 534)	50.5	3.2 (2.7; 3.6)	304 (201; 407)	71.8	2.0 (1.4; 2.7)
30-44	12,797,011	175 (96; 254)	32.2	1.4 (0.8; 2.0)	1044 (935; 1152)	32.3	8.2 (7.3; 9.0)	337 (274; 401)	36.3	2.6 (2.1; 3.1)	99 (69; 130)	23.5	0.8 (0.5; 1.0)
45-59	7,630,985	86 (55; 116)	15.8	1.1 (0.7; 1.5)	173 (95; 251)	5.4	2.3 (1.2; 3.3)	108 (76; 139)	11.6	1.4 (1.0; 1.8)	15 (15; 15)	3.5	0.2 (0.2; 0.2)
60+	4,878,530	30 (2; 58)	5.5	0.6 (0.0; 1.2)	33 (16; 51)	1.0	0.7 (0.3; 1.0)	15 (1; 29)	1.6	0.3 (0.0; 0.6)	5 (5; 5)	1.2	0.1 (0.1; 0.1)
Perpetrator race,[a] 15+ years (n= 5579)		567 (458; 675)			3521 (3198; 3844)			1034 (922; 1147)			456 (330; 583)		
African	31,385,166	436 (341; 531)	76.9	1.4 (1.1; 1.7)	2732 (2476; 2988)	77.6	8.7 (7.9; 9.5)	712 (618; 805)	68.8	2.3 (2.0; 2.6)	399 (284; 514)	87.4	1.3 (0.9; 1.6)
Indian/Asian	1,175,954	0	0	0.0 (0.0; 0.0)	10 (0; 24)	0.3	0.9 (0.0; 2.0)	0	0	0.0 (0.0; 0.0)	0	0	0.0 (0.0; 0.0)
Coloured	3,566,768	102 (78; 125)	17.9	2.9 (2.2; 3.5)	483 (448; 519)	13.7	13.5 (12.6; 14.6)	93 (55; 131)	9.0	2.6 (1.5; 3.7)	45 (28; 62)	9.9	1.3 (0.8; 1.7)
White	4,035,146	12 (0; 29)	2.1	0.3 (0.0; 0.7)	5 (5; 5)	0.1	0.1 (0.1; 0.1)	107 (70; 143)	10.3	2.7 (1.7; 3.5)	0	0	0.0 (0.0; 0.0)
Unknown	-	0	0	-	5 (5; 5)	0.1	-	9 (0; 21)	0.9	-	0	0	-
Foreign national	-	18 (9; 26)	3.1	-	286 (164; 407)	8.1	-	113 (70; 157)	11.0	-	13 (4; 21)	2.7	-
Victim perpetrator relationship (n= 5594)		567 (458; 675)			3531 (3208; 3854)			1039 (927; 1152)			456 (330; 583)		
Current wife (any legal or customary marriage)	-	37 (19; 54)	6.5	-	-	-	-	-	-	-	-	-	-
Current cohabitating girlfriend (no formal marriage)	-	167 (87; 246)	29.4	-	-	-	-	-	-	-	-	-	-
Current girlfriend (not living together)	-	10 (10; 10)	1.8	-	-	-	-	-	-	-	-	-	-
Ex-girlfriend	-	13 (4; 21)	2.2	-	-	-	-	-	-	-	-	-	-
Biological parent	-	38 (9; 67)	6.8	-	-	-	-	-	-	-	-	-	-
Biological child	-	10 (0; 24)	1.8	-	-	-	-	-	-	-	-	-	-
Sibling	-	126 (84; 169)	22.3	-	-	-	-	-	-	-	-	-	-
Other relative	-	80 (65; 95)	14.2	-	-	-	-	-	-	-	-	-	-
Stepparent	-	33 (8; 59)	5.9	-	-	-	-	-	-	-	-	-	-
Grand parent	-	5 (5; 5)	0.9	-	-	-	-	-	-	-	-	-	-
In laws	-	48 (31; 64)	8.4	-	-	-	-	-	-	-	-	-	-
Perpetrator romantically involved with victims current or ex-wife/girlfriend (love traingle)	-	-	-	-	40 (13; 67)	1.1	-	-	-	-	-	-	-
Neighbour	-	-	-	-	179 (122; 236)	5.1	-	-	-	-	-	-	-
Friend/ known by sight	-	-	-	-	2980 (2655; 3305)	84.4	-	-	-	-	-	-	-
Other	-	-	-	-	-	-	-	226 (181; 271)	21.8	-	-	-	-
Stranger	-	-	-	-	-	-	-	813 (703; 924)	78.2	-	-	-	-
Unknown	-	-	-	-	-	-	-	-	-	-	456 (330; 583)	100.0	
Perpetrator employment status, 15-64 years (n= 5040)**		507 (399; 614)			3203 (2922; 3485)			919 (813; 1025)			411 (290; 532)		
Employed	15,922,275	115 (82; 147)	22.6	0.7 (0.5; 0.9)	656 (544; 769)	20.5	4.1 (3.4; 4.8)	274 (201; 347)	29.8	1.7 (1.3; 2.2)	125 (78; 173)	30.5	0.8 (0.5; 1.1)
Unemployed	21,025,555	259 (191; 328)	51.1	1.2 (0.9; 1.6)	1818 (1601; 2035)	56.7	8.6 (7.6; 9.7)	461 (378; 543)	50.1	2.2 (1.8; 2.6)	161 (81; 240)	39.1	0.8 (0.4; 1.1)
Unknown	-	133 (81; 184)	26.1	-	729 (636; 823)	22.8	-	184 (138; 231)	20.0	-	125 (84; 167)	30.4	-
Perpetrator alcohol use (n= 5518)		562 (453; 670)			3498 (3174; 3822)			1024 (908; 1140)			434 (308; 560)		
Alcohol +ve	-	229 (156; 302)	40.7	-	1752 (1512; 1992)	50.1	-	203 (144; 263)	19.9	-	49 (15; 84)	11.3	-
Perpetrator other drug use (n= 5539)		567 (458; 675)			3501 (3186; 3817)			1032 (923; 1141)			439 (313; 565)		
Other drug +ve	-	15 (1; 29)	2.7	-	385 (317; 454)	11.0	-	77 (28; 125)	7.4	-	25 (8; 42)	5.7	-
Perpetrator with prior convictions		539 (428; 650)			3446 (3108; 3784)			1012 (894; 1130)			431 (306; 557)		
Perpetrator with prior convictions (n= 5429)	-	28 (19; 36)	5.1	-	405 (345; 465)	11.8	-	184 (126; 241)	18.1	-	45 (20; 69)	10.3	-
Multiple perpetrators		567 (458; 675)			3531 (3208; 3854)			1039 (927; 1152)			456 (330; 583)		
Multiple perpetrators (n= 5594)	-	8 (0; 16)	1.3	-	427 (351; 503)	12.1	-	226 (163; 288)	21.7	-	79 (36; 123)	17.3	-
Homicide part of gang related violence (n= 5102)		562 (453; 670)			3506 (3185; 3828)			1034 (922; 1147)			456 (330; 583)		
Yes	-	0	0	-	218 (188; 249)	6.2	-	45 (25; 65)	4.4	-	10 (10;10)	2.2	-
No	-	542 (429; 654)	96.4	-	3004 (2662; 3346)	85.7	-	919 (802; 1036)	88.9	-	401 (275; 528)	88.0	-
Unknown	-	20 (0; 48)	3.6	-	284 (216; 351)	8.1	-	70 (42; 98)	6.8	-	45 (45, 45)	9.9	-

* Age standardised rate per 100,000 population
** Excludes unknown ages
