# Supplementary material for: Who is killing South African men? A retrospective descriptive study of forensic and police investigations into male homicide
Source: BMJ Glob Health. 2024 Apr 10;9(4):e014912. doi: 10.1136/bmjgh-2023-014912 (PMC11015244; doi:10.1136/bmjgh-2023-014912)
Supplement: Supplementary data [file bmjgh-2023-014912supp003.pdf]

Table 3. Victim and perpetrator characteristics of male homicides killed by female and male perpetrators for selected covariates (n= 5571*)

	Female perpetrator n= 379 (6.8%)								Male perpetrator n= 5192 (93.2%)					
	Family				Acquaintance		Stranger/unknown relationship		Family		Acquaintance		Stranger/unknown relationship	
	Intimate partner		Other family						Other family					
	n (95% CI)	%	n (95% CI)	%	n (95% CI)	%	n (95% CI)	%	n (95% CI)	%	n (95% CI)	%	n (95% CI)	%
All male homicides 18+ years killed by known perpetrators (n= 5571)	226 (146; 306)	59.6	25 (11; 39)	6.6	113 (49; 176)	29.8	15 (15; 15)	4.0	316 (260; 372)	6.1	3408 (3111; 3706)	65.6	1468 (1276; 1660)	28.3
Victim age group (n= 5551)	226 (146; 306)		25 (11; 39)		113 (49; 176)		15 (15; 15)		316 (260; 372)		3398 (3101; 3696)		1458 (1265; 1652)	
18-29	57 (32; 82)	25.1	15 (1; 29)	60.0	65 (26; 105)	57.9	0 (0; 0)	0.0	131 (103; 159)	41.4	1778 (1617; 1939)	52.3	742 (623; 860)	50.9
30-44	124 (51; 197)	55.0	5 (5; 5)	20.0	18 (9; 26)	15.5	10 (10; 10)	66.7	103 (64; 142)	32.6	1254 (1086; 1421)	36.9	510 (413; 607)	35.0
45-59	30 (30; 30)	13.3	5 (5; 5)	20.0	20 (0; 48)	17.7	0 (0; 0)	0.0	72 (31; 114)	22.9	257 (195; 319)	7.6	124 (68; 179)	8.5
60+	15 (15; 15)	6.7	0 (0; 0)	0.0	10 (10; 10)	8.9	5 (5; 5)	33.3	10 (0; 24)	3.2	110 (74; 146)	3.2	83 (56; 109)	5.7
External cause (n= 5551)	226 (146; 306)		25 (11; 39)		113 (49; 176)		15 (15; 15)		316 (260; 372)		3408 (3111; 3706)		1468 (1276; 1660)	
Sharp force	189 (109; 270)	83.8	25 (11; 39)	100.0	100 (37; 163)	88.9	10 (10; 10)	66.7	244 (183; 304)	77.1	2430 (2162; 2698)	71.3	772 (614; 931)	52.6
Gunshot	12 (0; 29)	5.2	0 (0; 0)	0.0	0 (0; 0)	0.0	0 (0; 0)	0.0	44 (10; 78)	13.9	511 (436; 585)	15.0	541 (452; 629)	36.8
Blunt	15 (1; 29)	6.7	0 (0; 0)	0.0	13 (4; 21)	11.1	5 (5; 5)	33.3	28 (7; 49)	9.0	264 (208; 320)	7.7	57 (23; 90)	3.9
Other	10 (10; 10)	4.4	0 (0; 0)	0.0	0 (0; 0)	0.0	0 (0; 0)	0.0	0 (0; 0)	0.0	204 (132; 276)	6.0	99 (73; 124)	6.7
Victim race (n= 5566)	226 (146; 306)		25 (11; 39)		113 (49; 176)		15 (15; 15)		316 (260; 372)		3403 (3106; 3701)		1468 (1276; 1660)	
African	143 (81; 206)	63.5	10 (0; 24)	40.0	98 (34; 161)	86.7	10 (10; 10)	66.7	281 (225; 337)	88.9	2838 (2538; 3137)	83.4	1286 (1099; 1473)	87.6
Indian/Asian	9 (0; 21)	4.0	0 (0; 0)	0.0	0 (0; 0)	0.0	0 (0; 0)	0.0	0 (0; 0)	0.0	50 (36; 64)	1.5	15 (1; 29)	1.0
Coloured	62 (38; 85)	27.3	15 (15; 15)	60.0	15 (15; 15)	13.3	5 (5; 5)	33.3	35 (35; 35)	11.1	500 (434; 566)	14.7	115 (83; 147)	7.8
White	12 (0; 29)	5.2	0 (0; 0)	0.0	0 (0; 0)	0.0	0 (0; 0)	0.0	0 (0; 0)	0.0	0 (0; 0)	0.0	52 (27; 78)	3.6
Victim employment status (n= 5477)	226 (146; 306)		25 (11; 39)		113 (49; 176)		15 (15; 15)		316 (260; 372)		3356 (3060; 3651)		1426 (1218; 1634)	
Employed	77 (42; 112)	34.3	0 (0; 0)	0.0	15 (1; 29)	13.3	0 (0; 0)	0.0	35 (15; 56)	11.2	473 (379; 568)	14.1	237 (190; 284)	16.6
Unemployed	48 (19; 78)	21.4	15 (15; 15)	60.0	40 (17; 63)	35.8	10 (10; 10)	66.7	146 (89; 204)	46.3	1577 (1350; 1803)	47.0	453 (361; 544)	31.7
Unknown	100 (62; 138)	44.3	10 (0; 24)	40.0	58 (0; 115)	51.0	5 (5; 5)	33.3	134 (93; 176)	42.5	1306 (1151; 1461)	38.9	737 (586; 888)	51.7
Setting (n= 5571)	226 (146; 306)		25 (11; 39)		113 (49; 176)		15 (15; 15)		316 (260; 372)		3408 (3111; 3706)		1468 (1276; 1660)	
Urban formal	23 (3; 44)	10.3	0 (0; 0)	0.0	43 (8; 77)	37.7	5 (5; 5)	33.3	73 (43; 102)	23.0	813 (680; 946)	23.9	542 (465; 619)	36.9
Urban informal	107 (70; 145)	47.6	10 (10; 10)	40.0	60 (28; 93)	53.3	5 (5; 5)	33.3	77 (40; 115)	24.5	1082 (935; 1230)	31.8	457 (359; 556)	31.1
Rural	95 (47; 143)	42.1	15 (1; 29)	60.0	10 (0; 24)	8.9	5 (5; 5)	33.3	166 (112; 220)	52.6	1512 (1271; 1754)	44.4	469 (328; 610)	31.9
Place of homicide (n= 5475)	226 (146; 306)		25 (11; 39)		113 (49; 176)		15 (15; 15)		316 (260; 372)		3378 (3069; 3688)		1402 (1208; 1595)	
Victim home	191 (115; 267)	84.5	15 (1; 29)	60.0	20 (20; 20)	17.7	0 (0; 0)	0.0	245 (189; 301)	77.6	396 (309; 483)	11.7	177 (116; 238)	12.6
Perpetrator home	0 (0; 0)	0.0	5 (5; 5)	20.0	30 (0; 62)	26.6	5 (5; 5)	33.3	0 (0; 0)	0.0	62 (31; 93)	1.8	62 (23; 100)	4.4
Someone else home	8 (0; 16)	3.3	0 (0; 0)	0.0	0 (0; 0)	0.0	0 (0; 0)	0.0	15 (1; 30)	4.9	270 (191; 348)	8.0	85 (53; 117)	6.1
Public space (road, park, shop,mall)	8 (8; 0; 16)	3.3	5 (5; 5)	20.0	63 (25; 100)	55.7	10 (10; 10)	66.7	45 (13; 77)	14.4	1651 (1464; 1838)	48.9	848 (704; 993)	60.5
Recreational setting (bar, shebeen)	20 (0; 40)	8.9	5 (5; 5)	0.0	0 (0;0)	0.0	0 (0; 0)	0.0	10 (10; 10)	3.2	805 (672; 938)	23.8	93 (63; 122)	6.6
Other	0 (0; 0)		0 (0; 0)	0.0	0 (0; 0)	0.0	0 (0; 0)	0.0	0 (0; 0)	0.0	194 (147; 242)	5.8	138 (96; 176)	9.8
Perpetrator characteristics														
Perpetrator age group, 15+ years (n= 5115)	221 (141; 301)		25 (11; 39)		73 (37; 109)		15 (15; 15)		298 (245; 352)		3150 (2877; 3423)		1333 (1147; 1518)	
15-29	90 (46; 134)	40.8	10 (10; 10)	40.0	45 (10; 81)	62.3	5 (5; 5)	33.3	153 (106; 200)	51.3	1938 (1742; 2134)	61.5	763 (627; 899)	57.3
30-44	96 (25; 167)	43.4	0 (0; 0)	0.0	28 (19; 36)	37.7	10 (10; 10)	66.7	79 (40; 119)	26.6	1006 (895; 1116)	31.9	427 (353; 501)	32.0
45-59	30 (30; 30)	13.6	10 (0; 24)	40.0	0 (0; 0)	0.0	0 (0; 0)	0.0	46 (19;73)	15.4	173 (95; 251)	5.5	123 (91; 154)	9.2
60+	5 (5; 5)	2.3	5 (5; 5)	20.0	0 (0; 0)	0.0	0 (0; 0)	0.0	20 (0; 48)	6.7	33 (16; 51)	1.1	20 (6; 34)	1.5
Perpetrator race, 15+ years (n= 5571)	226 (146; 306)		25 (11; 39)		113 (49; 176))		10 (10; 10)		316 (260; 372)		3398 (3101; 3696)		1468 (1276; 1660)	
African	150 (88; 212)	66.4	10 (0; 24)	40.0	78 (38; 118)	69.0	5 (5; 5)	50.0	276 (220; 332)	87.3	2644 (2390; 2898)	77.8	1098 (926; 1271)	74.8
Indian/Asian	0 (0; 0)	0.0	0 (0; 0)	0.0	0 (0; 0)	0.0	0 (0; 0)	0.0	0 (0; 0)	0.0	10 (0; 24)	0.3	0 (0; 0)	0.0
Coloured	52 (28; 75)	22.9	15 (15; 15)	60.0	10 (10; 10)	8.9	5 (5; 5)	50.0	35 (35; 35)	11.1	473 (438; 509)	13.9	128 (87; 170)	8.8
White	12 (0; 29)	5.2	0 (0; 0)	0.0	0 (0; 0)	0.0	0 (0; 0)	0.0	0 (0; 0)	0.0	5 (5; 5)	0.1	107 (70; 143)	7.3
Unknown	0 (0; 0)	0.0	0 (0; 0)	0.0	0 (0; 0)	0.0	0 (0; 0)	0.0	0 (0; 0)	0.0	5 (5; 5)	0.1	9 (0; 21)	0.6
Foreign national	13 (4; 21)	5.5	0 (0; 0)	0.0	25 (0; 53)	22.2	0 (0; 0)	0.0	5 (5; 5)	1.6	261 (165; 356)	7.7	126 (77; 175)	8.6
Perpetrator alcohol use (n= 5496)	221 (141; 301)		25 (11; 39)		113 (49; 176)		15 (15; 15)		316 (260; 372)		3375 (3077; 3672)		1431 (1228; 1634)	
Alcohol +ve	118 (68; 167)	53.3	10 (10; 10)	40.0	50 (0; 107)	44.3	0 (0; 0)	0.0	101 (70; 132)	32.0	1702 (1498; 1906)	50.4	253 (180; 325)	17.6
Perpetrator other drug use (n= 5506)	226 (146; 306)		25 (11; 39)		113 (49; 176)		15 (15; 15)		316 (260; 372)		3378 (3090; 3666)		1433 (1252; 1634)	
Other drug +ve	0 (0; 0)	0.0	0 (0; 0)	0.0	0 (0; 0)	0.0	0 (0; 0)	0.0	15 (1; 29)	4.8	385 (317; 454)	11.4	102 (50; 153)	7.1
Perpetrator with prior convictions (n= 5406)	218 (140; 297)		25 (11; 39)		113 (49; 176)		15 (15; 15)		296 (233; 359)		3323 (3015; 3631)		1416 (1221; 1610)	
Perpetrator with prior convictions	0 (0; 0)	0.0	0 (0; 0)	0.0	0 (0; 0)	0.0	0 (0; 0)	0.0	28 (19; 36)	9.3	405 (345; 465)	12.2	223 (161; 286)	15.8

* Excludes 23 cases with unknown perpetrator gender
